# Mercury contamination and human exposure through inland fishery and aquaculture products in Northeastern Brazil

**DOI:** 10.1007/s10661-026-15512-w

**Published:** 2026-06-02

**Authors:** Luiz Drude de Lacerda, Moisés Fernandes Bezerra

**Affiliations:** Laboratório de Biogeoquímica Costeira, Instituto de Ciências Do Mar, Universidade Federal Do Ceará, Av. Abolição 3207, 60.165-081 Fortaleza, Brazil

**Keywords:** Mercury contamination, Fish farming, Risk assessment, Inland waters

## Abstract

The exponential growth of inland fisheries and aquaculture in Northeastern Brazil requires permanent monitoring of the quality of their products, particularly regarding persistent globally distributed pollutants such as mercury (Hg). This is the first survey on Hg concentrations in inland fishery and aquaculture products from the region. This survey includes an inventory of Hg concentrations vis-à-vis compliance with national and international food safety guidelines and an evaluation of Hg exposure risk from fishery and aquaculture products consumption. Mercury concentrations were quantified in 14 fish species, representing the regional inland fisheries and including the main aquaculture product, the Tilapia (*Oreochromis niloticus*). Overall, concentrations were very low and well below the established threshold for human and environmental health protection. As expected, concentrations were higher in carnivorous species, such as *Serrasalmus rhombeus* (Piranha) from fluvial systems. We found an important pattern of Hg accumulation in farmed Tilapia, where market-size fish presented lower Hg concentrations than juveniles. This pattern likely reflects accelerated growth under intensive feeding conditions, resulting in dilution of Hg within rapidly increasing biomass. Estimates of Hg ingestion by fish consumers indicate very low exposure risk, even for high consumption scenarios, suggesting that consumption of these fishery and aquaculture products is safe with respect to Hg exposure.

## Highlights

• Mercury concentrations are much lower than legal limits for fishery and aquaculture products in NE Brazil.

• Aquaculture fish shows decreasing Hg concentrations with size, and lowest Hg contents in commercial-size individuals.

• Fish from river habitats showed higher Hg than those of the same species but sampled in reservoirs.

• Wild fish from rivers show Hg biomagnification, with a positive size-Hg relationship.

• No significant human risk to Hg exposure through consumption of fish from extractive fishery or aquaculture in NE Brazil.

**Graphical Abstract:**

## Graphical abstract

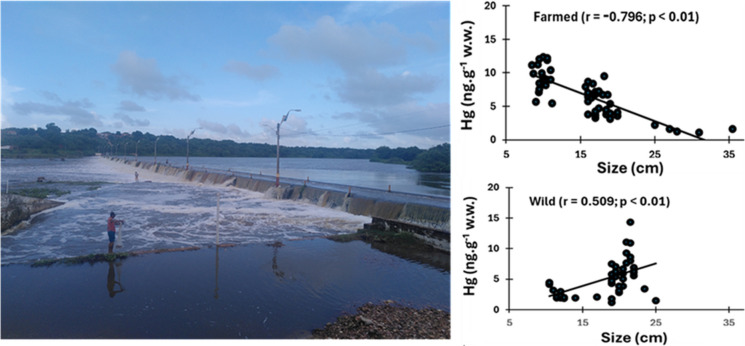

## Introduction

The contamination of fish and aquaculture products can become an obstacle to the achievement of the economic potential of the sector in northeastern Brazil and represents, if evidenced, a source of risk to public health and food safety. Future scenarios of exposure to contaminants, particularly for those with persistent behavior and global distribution, are urgent as a way to subsidize measures to improve the sustainability, in the short and medium terms, of the fishing sector. Considering the exponential growth of inland fisheries and aquaculture in the region, these measures are necessary to improve the sector and the state government’s responses to eventual issues raised in fish quality audits, as production outflow can be impacted if banning measures to the commercialization of fish and aquaculture products are put in place (Bezerra et al., 2023).

Mercury (Hg) is a metal that is toxic to humans and other organisms. It occurs naturally in the environment in the form of metallic or elemental Hg (Hg^0^), inorganic (Hg^2+^) and forming organic complexes (e.g., CH_3_Hg^+^) and is considered a global, persistent pollutant (Colombo et al., 2004; UN Environment, 2019). The northeastern region of Brazil does not have significant natural sources of Hg, such as cinnabar mineral deposits and/or volcanic activities. However, anthropogenic activities, such as the burning of fossil fuels, effluents from industries, urban areas, agriculture and aquaculture, can contribute to the emission of Hg into the environment, including the atmosphere, water, sediment, and biota, whereas regional land use changes and global warming are increasing Hg load to aquatic ecosystems throughout the region (Lacerda et al., 2020; Luz-Santos et al., 2023).

The most toxic form of Hg is methyl-Hg (CH_3_Hg^+^), an organic form produced by decomposer organisms in sediment and/or water column, which has the capacity for bioaccumulation in organisms and biomagnification in the trophic chain. Methyl-Hg can be concentrated in organisms at the top of the food chain at levels up to 10,000 times higher than those found in water and sediment (UN Environment, 2019). Therefore, seafood consumption becomes the main route of exposure to methyl-Hg in humans, despite its known nutritional benefits to the general human populations. Excessive human exposure to Hg can cause nervous system problems, especially in sensitive populations such as women of childbearing age, newborns and children (Harada, 1995). Aiming to reduce the risks of health problems associated with consumption of Hg-contaminated fish, health authorities have developed recommendations that establish a tolerable weekly intake (TWI) (1.3 µg.kg^−1^ body weight) and upper-level limits for Hg content in food, including fish (FAO/WHO, 2010) . These recommendations are widely used in the regulation and legislation of the fishing and aquaculture industries in Brazil and worldwide.

Historically, in the semi-arid climate region of northeastern Brazil, fish production and artisanal fishing in artificial reservoirs and lakes are important activities that provides subsistence food and income to the local population. The fishing of these water bodies with exotic and native species has been a recurrent practice in the northeast region as an alternative to low agricultural productivity and to provide an improvement in the diet of local populations. Among the main species used in this fishing practice are the Curimatã (*Prochilodus brevis*), the Piau (*Leporinus* sp.), Traíras (*Hoplias malabaricus* and *H. brasiliensis*), Tilapia (*Oreochromis niloticus* and *Tilapia rendalli*), Hake (*Plagiossion squamossimus*), and Peacock Bass (*Cichla* sp.), among others. Apart from extractive fisheries, Tilapia aquaculture from the northeastern region responded with 86,000 tones, approximately 17% of Brazil’s production of nearly 500,000 tons, in 2024 (IBGE, 2025). Therefore, considering the importance of these species as subsistence for populations inhabiting regions surrounding public reservoirs and rivers in the region, it is important to estimate human exposure to Hg through the consumption of these inland water fish species, similar to those already existing from marine fisheries (Bezerra et al., 2023).

This study presents, for the first time, an inventory of Hg concentrations in inland waters fish species, from extractive fisheries and aquaculture, of dietary and economic importance to the local population of Ceará, Brazil. Furthermore, the study presents the first estimates of Hg exposure through freshwater fish consumption in northeastern Brazil and proposes aquaculture strategies to minimize Hg bioaccumulation.

## Material and methods

### Sampling and Hg quantification

Mercury (Hg) concentrations were quantified in 14 fish species typical representatives of continental fisheries in northeastern Brazil (Table 1). For three species, *Hoplias malabaricus* (Traíra), *Plagioscion squamosissimus* (Hake) and *Serrasalmus rhombeus* (Piranha), individuals were sampled from reservoirs and rivers, while for *Oreochromis niloticus* (tilapia), individuals were sampled from fish farms and open reservoirs. Total Hg concentrations were quantified in a total of 245 individuals while methyl-Hg concentrations were quantified in 43 individuals, from 8 species. For two species (e.g. *H. malabaricus* (Traíra) and *Leporinus friderici* (Piau)), the sample size was insufficient (< 5 individuals) to represent the species and, therefore, the results should be interpreted with caution. Fish Hg concentrations are reported in ng.g^−1^ of wet weight.

**Table 1:** Species, common name, total Hg concentrations, size and the percentage of methyl-Hg (mean, standard deviation and size range) and habitat (Re = reservoir; Fl = fluvial) in freshwater fish from inland waters in NE Brazil

Species	Common name	Total Hg (ng g^−1^ w.w.)	LT (cm)	*n*	Methyl-Hg (%)	*n*
*Astronotus ocellatus* (Re)	Oscar fish	14.9 ± 7.9 (6.1–29.4)	25.4 ± 0.8 (24.5–27.0)	7	89.7 (88.1–91.3)	2
*Cichla kelberi* (Re)	Peacock Bass	8.4 ± 4.4 (3.3–22.7)	29.4 ± 3.6 (20.0–35.5)	27	61.4 (44.5–87.6)	3
*Cichla pinima* (Re)	Peacock Bass	6.8 ± 2.4 (4.2–12.9)	27.2 ± 2.8 (22.5–33.5)	17	88.1 (72.1–100)	3
*Cichla* sp. (Fl)	Peacock Bass	28.4 ± 12.8 (13.3–59.5)	25.3 ± 2.5 (21.5–29.0)	10	–	-–
*Hoplias malabaricus* (Fl)	Traíra	21.1	33	1	79.2	1
*H. malabaricus* (Re)	Traíra	6.8 ± 1.4 (5.2–7.7)	25.7 ± 3.7 (25.0–26.0)	3	71.6 (58.0–78.6)	3
*Hoplosternum littorale* (Fl)	Catfish	17.8 ± 7.8 (4.9–28.6)	21.7 ± 2.7 (18.0–26.0)	6	–	–
*Hypostomus pusarum* (Fl)	Catfish	4.3 ± 1.1 (2.9–5.6)	20.2 ± 0.9 (19.0–21.0)	8	–	–
*Oreochromis niloticus* (Re-Wild)	Tilapia	5.1 ± 2.9 (1.2–14.3)	18.3 ± 4.1 (10.5–25.0)	44	71.9 (49.7–95.2)	19
*O. niloticus* (Re-Farmed)	Tilapia	6.6 ± 3.0 (1.1–12.4)	15.9 ± 5.9 (8.5–33.5)	56	84.5 (52.4–100)	18
*Leporinus friderici* (Re)	Piau	26.1 ± 14.0 (12.6–49.5)	20.9 ± 1.1 (18.5–23.0)	4	–	–
*Plagioscion squamosissimus* (Fl)	Hake	29.6–18.0 (10.8–49.5)	25.2–2.9 (22.5–29.0)	4	–	–
*P. squamosissimus* (Re)	Hake	2.6	17.5	1	54.8	1
*Prochilodus argenteus* (Fl)	Curimata	6.2 ± 1.5 (3.2–8.4)	20.2 ± 4.5 (19.0–31.0)	9	94.5 (81.7–100)	5
*P. brevis* (Re)	Curimata	8.1 ± 4.0 (8.0–18.8)	19.3–1.5 (18.5–23.5)	12	–	–
*P. rubrotaeniatus* (Fl)	Curimata	11.5 ± 5.0 (5.7–21.6)	25.3 ± 3.2 (16.5–29,5)	12	–	–
*Serrasalmus rhombeus* (Fl)	Piranha	40.9 ± 24.8 (7.6–68.5)	17.4 ± 2.4 (13.3–21.0)	10	100	5
*Serrasalmus rhombeus* (Re)	Piranha	10.9 ± 6.7 (3.0–27.1)	18.1 ± 2.0 (15.5–23,5)	14	67.0 (35.0–93.0)	3

**Fig. 1:**
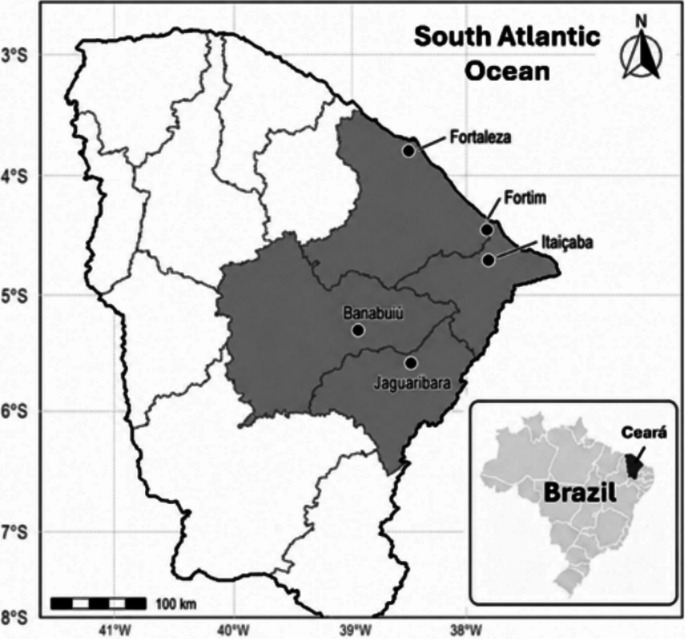
Map showing the locations of sampling campaigns in Ceará State. Gray-shaded areas denote the sampled watershed basins and black dots are municipalities where fish markets were visited

Fish samples were obtained from major reservoirs and rivers from the state of Ceará in northeastern Brazil (Fig. 1) between 2013 and 2023 and samplings were not designed to assess seasonal variations. All sampling sites occur in the larger Jaguaribe River basin; therefore, they are somewhat linked directly or through river diversions typical of the semiarid region.

Species were acquired directly from fishermen and chosen based on surveys in local markets and fishers’ associations on their relative commercial importance of greatest interest in terms of fish consumption. Fish identification was based on the catalog from Botero et al. (2023) that describes the continental aquatic fauna of the state of Ceará. Samples were collected directly in rivers and reservoirs using traditional fishery techniques. When available, samples were obtained in points of sale and consisted only of fresh individuals and without any type of treatment. The samples were packaged and preserved in Styrofoam with ice until they arrived at the laboratory for further processing.

All the glassware and materials used for the treatment and digestion of the samples were previously washed in a neutral detergent bath followed by immersion for 24 h in a 10% Hg-free HCl solution. Only fish muscle tissue was used, since it is the part normally consumed, both by predators and by the human population. From 0.5 to 2.0 g of the lyophilized sample were weighed directly in metal-free Teflon® tubes. Then, 10 mL of concentrated nitric acid (HNO_3_) were added to each tube and digested in a microwave oven CEM MARs-MD 1744 at 200 °C for 30 min, after digestion, 1 mL of hydrogen peroxide (H_2_O_2_) to avoid Hg re-complexation The concentrations of Hg were quantified by cold vapor atomic absorption spectrophotometry (CV-AAS, NIPON® NIC RA-3), after Hg reduction with NaBH_4_ (Lacerda et al., 2014; Moura & Lacerda, 2018, 2022). The limit of detection (LOD) was calculated according to USEPA (2000) as the standard deviation of 7 blank replicates multiplied by 3 and was 0.2 ng.g^−1^ Simultaneously, certified standards ERMBB422—Fish Muscle were analyzed and gave average recoveries varying from 88 to 101%. The limit of quantification (LOQ) of the procedure was the LOD multiplied by 10 (USEPA, 2000) and was 1.0 ng.g^−1^. Concentration values obtained were not corrected for the relative recoveries obtained for the certified materials. Quantification of methyl-Hg was performed in duplicate with 100 mg of lyophilized muscle samples weighed in PTFE tubes. Five (5) mL of 25% KOH were added in each tube and transferred to an oven at 70 °C for 6 h, gently agitating them every hour. Samples were kept in a dark environment to prevent methyl-Hg degradation. For sample ethylation it was added to each tube 300 μL of a 2 mol.L^−1^ acetate buffer (pH 4.5), 30 μL of each sample, and 50 μL of 1% sodium tetraethyl borate. Ethylated extracts were taken to a final volume of 40 mL by adding ultrapure water (Milli-Q). Methyl-Hg concentrations were obtained by gas chromatography coupled with an atomic fluorescence spectrometer (GC-AFS–MERX-TM Brooks Rand automated methyl-Hg system. Certified reference material (Tuna Fish-BCR-463) analyzed using the same procedure yielded an average recovery of 82%. The average LOD was 0.02 ng.g^−1^ and LOQ was 0.25 ng.g^−1^. The analyses were conducted at the WCP Environmental Biogeochemistry Laboratory, Federal University of Rondônia (UNIR). The procedures for collection, sample processing, and data processing follow recommendations from USEPA (2000, 2001, 2022) reviewed in detail and adapted to the regional situation in Barbosa et al. (2021) and Caracas et al. (2022).

### Estimation of Hg exposure for the different scenarios of fish consumption

The estimated exposure for the different scenarios of fish consumption was calculated according to internationally accepted procedures (USEPA, 2000; 2022). Exposure risk estimates considered total Hg concentrations as this is the easiest and most common way Hg data is reported in fish, and we conservatively assumed that all Hg present in fish as methylmercury and thus using a precaution approach more protective to human health. Nevertheless, we still report methylmercury average values and percentages for most of the studied species.

The *Fish Safe Level* (FSL), which is an a priori maximum safe screening level (Eq. 1), was calculated using the average body weight (MBW), in kg, for each consumer group and the per capita fish consumption (FC) considering the three consumption scenarios: a) FC_NE_ (Per capita fish consumption in NE Brazil) = 0.0245 kg.day^−1^ (IBGE, 2021); b) FC_WHO_ (WHO recommended fish consumption) = 0.0285 kg.day^−1^ (FAO/WHO, 2010; USDA, 2010); and c) FC_SUBS_ (subsistence/high fish consumption) = 0.142 kg.day^−1^ (USEPA, 2001). The reference dose (Rfd) for Hg of 0.0001 mg.kg body weight^−1^·day^−1^ (USEPA, 2001) is defined as the level of daily Hg exposure at which adverse effects are not expected. The average body weight (MBW) varies according to the different groups of consumers; women of childbearing age (18 to 45 years, 58.2 kg); children (1 to 12 years, 23.8 kg); adult men (over 18 years old, 68.3 kg); and adult women (over 46 years old, 59.8 kg) (IBGE, 2010).

1 $$FSL= \frac{MBW \times RfD}{FC}$$

We calculated the maximum safe fish intake (FCmax), in kg.day^−1^, using the parameters MBW, RfD and the average Hg concentration measured in the fish ([Hg]_fish_), in mg.kg^−1^: (Eq. 2).

2 $${FC}_{max}= \frac{MBW \times RfD}{{[Hg]}_{fish}}$$

The estimated daily ingestion (EDI) was calculated in mg.kg body weight^−1^·day^−1^ for each group of consumers at the three consumption scenarios (Eq. 3) and was compared to the reference dose for Hg (RfD).

3 $$EDI= \frac{{[Hg]}_{fish} \times FC}{MBW}$$

The *Target Hazard Quotient* (THQ) was estimated through Eq. 4 (USEPA, 2022). This index represents the risk of chronic exposure to non-carcinogenic contaminants, where a THQ < 1 means no potential risk of adverse effects and for THQ > 1 there is a potential risk of adverse effects in consumers.

4 $$THQ= \frac{{FE \times FC \times DE \times [Hg]}_{fish}}{RfD \times MBW \times FE \times DE}$$

where FE is the frequency of exposure (365 day·year^−1^) and DE is the duration of exposure (77 years for adults and 12 years for children). This index was evaluated for different scenarios of fish consumption (FC).

To obtain the recommended safe monthly fish meals (FC_meals_) for each group of consumers, the maximum safe consumption rate (FC_max_) was previously calculated, and the estimated duration of exposure (DE) was used, defined as 365.25 days in 12 months or 30.44 days per month. The portion size was defined as 150 g for adults and 75 g for children (FAO/WHO, 2010) (Eq. 5).

5 $${FC}_{meals}= \frac{{FC}_{max} \times DE}{portion size}$$

To simplify the recommendations provided in the present study, four categories of frequency of consumption were used for each species of fish evaluated: (1) no more than 3 meals per month; (2) 4 to 6 meals per month (or no more than one meal per week); (3) 8 to 10 meals per month (or no more than two meals per week); and (4) 11 to 13 meals per month (or no more than three meals per week). Species in which the recommendation is above 13 meals per month were defined as of no restriction to consumption.

### Data processing and statistical analysis

Data processing and statistical analyses were performed in R version 4.2.3 (2023-03−15 ucrt) using RStudio (2022.7.1.554). Normality assumptions for Hg concentration dataset were tested using Shapiro-Wilks (SW) test and comparisons among factors (e.g., species and location) were conducted using the non-parametric Kruskal–Wallis (KW) test. Following a significant KW test, a post-hoc multiple comparisons Dunn’s test, with P adjusted Benjamin-Hochberg method, was conducted to determine significant differences in Hg concentrations across factors. Spearman’s correlation tests were used to verify the relationship between Hg levels and fish size. Statistical significance was set at 0.05.

## Results and discussion

### Mercury (Hg) and methyl-Hg concentrations in freshwater fish

The different types of fish were grouped by species and source of collection, and the mean Hg concentration values per species were used to calculate exposure estimates. Table 1 presents the means, standard deviations, medians, IQRs and minimum and maximum concentrations of Hg and methyl-Hg obtained for each fish species by collection area. All species, collected in all localities, presented small to medium-sized individuals < 40 cm, and show rather narrow size ranges. The individuals of all species analyzed showed low Hg concentrations (< 70 ng.g^−1^ w.w.), well below the maximum limits allowed by the legislation 1,000 and 500 ng.g^−1^ w.w., for carnivorous fish and other species, respectively (ANVISA, 2021).

Concentrations were highest in carnivorous S. *rhombeus* (Piranha), commonly consumed by human populations locally, sampled from the Jaguaribe river (68.5 ng.g^−1^ w.w.). In all other carnivorous species, including *S. rhombeus* (Piranha) sampled from the Castanhão Reservoir, concentrations were much lower (< 30 ng.g^−1^ w.w.) and did not differ significantly (*p* > 0.05) from herbivorous and omnivorous species. Overall, the observed concentrations are similar to those reported for the northeastern regions (Lacerda et al., 2014), but much lower than values reported for other regions in Brazil. In the Amazon region, Hg concentrations are one order of magnitude higher (Bastos et al., 2015, and references therein). Average methyl-Hg fraction relative to total Hg content varied from 54.8% in *P. squamosissimus* (Hake) from the Castanhão Reservoir to 100% in S. *rhombeus* (Piranha) sampled from the Jaguaribe river, and again with the exception of these last individuals, no significant difference (*p* > 0.05) in methyl-Hg proportions were found among species, irrespective of feeding.

No significant differences (*p* > 0.05) were observed between the collection sites for most species, with the exception of the top carnivores: *H. malabaricus*, *P. squamosissimus*, and *S. rhombeus*; however, only *S. rhombeus* provided a sufficient number of samples to statistically test this difference and this species presented significantly higher (p < 0.01) average Hg concentrations (40.9 ng.g^−1^ w.w.) and methyl-Hg fraction (100%) in the Jaguaribe River, downstream the Castanhão Reservoir, compared to the same species from the reservoir itself (10.9 ng.g^−1^ w.w. and 67%). Fish size from both sites was not significantly different (*p* > 0.05). While *S. rhombeus* is considered a top predator, it is rather an opportunistic species generally feeding on sick, injured, or dead fish, but also including insects, crustaceans, and live small fish, occasionally even small amphibians, reptiles, or mammals in its diet (Goulding, 1980).

We found very scarce literature comparing the diet of *S. rhombeus* in lotic and lentic environments, mostly from tropical America, where the species is endemic. Mol et al. (2007) compared the food habits of the fish fauna in the Brokopondo Reservoir to those downstream in the Suriname River, prior to dam construction. Fish species reduced from 172 in the river to only 41 in the reservoir. In addition, reservoir fishes showed lower species diversity and evenness and a dominance of only a few species. Fishes in the Brokopondo Reservoir are mostly small-sized species and individuals compared to the Suriname River before the closure of the dam. Thus, in the reservoir, *S. rhombeus* prey on shoals of small pelagic *Bryconops* spp. and shows reduced standard length as compared to riverine populations. Reservoir *S. rhombeus* also fed to some extent on food from the forest. Notwithstanding, *S. rhombeus* from the Brokopondo Reservoir-Suriname River presents much higher Hg concentrations of up to 920 ng.g^−1^ (converted from original dry weight concentrations) compared to our results from northeastern Brazil, due to intensive artisanal gold mining in the watershed (Mol et al., 2001). Therefore, the presence of this significant source of Hg in the Suriname sites and the semiarid vegetation surrounding the Castanhão Reservoir create completely different environmental conditions and hampers a direct comparison with the Hg concentrations found in Northeastern Brazil.

*Serrasalmus* sp., a different piranha species, showed Hg concentrations varying from 224 to 359 ng.g^−1^ w.w. in non-contaminated rivers in São Paulo State, whereas in the non-contaminated Salto Grande reservoir, concentrations were lower (96 ± 55 ng.g^−1^ w.w.), although in the contaminated Jurumirim Reservoir, that showed high methyl-Hg content in sediment and water, concentrations were much higher (Tomazelli et al., 2007). This pattern is similar to that observed in *S. rhombeus*, with lower Hg concentrations in reservoir fish compared to rivers.

In the southern Amazon Machado river, *S. rhombeus* feeds mainly on fish (> 80%), particularly on *Bricon* spp. (Goulding, 1980), a larger prey than *Bryconops* spp. the typical prey from reservoirs (Mol et al., 2007). In Venezuelan flood plains, juvenile *S. rhombeus* are specialized on preying fish fins, although aquatic insects, were also present. Larger specimens present whole small fish, chunks of fish flesh, and fish fins (Nico & Taphorn, 1988). In the São Miguel river, in western Amazon, *S. rhombeus* are mostly piscivorous and its diet depends on prey availability and predator to prey size ratio. An spatial pattern was evidenced in that river, with fish caught in the river headwaters being entire piscivorous (100%, both in frequency and relative weight of food items), whereas in intermediate sector and the mouth of the river plant material and worms and larvae contribute with 33.3% and 2.1% frequency, respectively, although these two items sum up only 2.7% in weight among food items (Bezerra-Neto et al., 2024). The highest proportion of methyl-Hg in the fish sampled from the Jaguaribe river, downstream the Castanhão Dam, suggests they prey on higher trophic levels fish and thus with higher Hg concentrations, relative to the individuals sampled inside the reservoir that feed lower in the food web, thus with lower Hg concentrations. Hylander et al. (2000) related higher Hg concentrations in *Serrasalmus* sp. during the low water period in the Pantanal wetlands in central Brazil and suggested that the high concentrations associated with decreasing water volumes could be an effect of habitat contraction potentializing predation, and consequently higher Hg bioaccumulation.

Dórea et al. (2004) also associated higher Hg concentrations in the dry season with lower pH in the Rio Negro Basin in the Amazon, since lower pH favors Hg methylation, increasing Hg bioavailability. Unfortunately, there is no study comparing the two populations of *S. rhombeus* along the reservoir-river continuum in northeastern Brazil, but average pH of the Castanhão reservoir from 2011 to 2014 including dry and wet seasons was 8.05 (7.5–8.7) (Lacerda et al., 2018), whereas the Jaguaribe waters are slightly more acidic; 7.1 (6.6–7.8), also from a period covering dry and wet seasons of 2006 and 2007 (Molisani et al., 2013). Therefore, Hg methylation and thus bioavailability would be higher in the riverine environment compared to the reservoir. In addition, the Jaguaribe River volume is drastically reduced during the dry season and may result in the increasing prey concentration favoring predation, as hypothesized for the Pantanal wetlands (Hylander et al., 2000). These aspects may be related to the higher concentrations found in the fluvial population compared to that of the reservoir. The results (Table 1) showed that the other top carnivores may display the same pattern and suggest an interesting hypothesis to text, although the small number of samples impairs a better discussion.

**Fig. 2:**
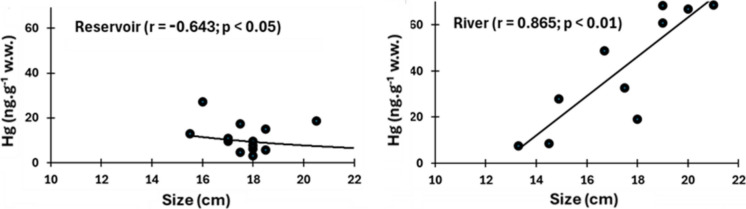
Relationship between size and Hg concentrations in *S. rhombeus* sampled in the Castanhão Reservoir and downstream in the Jaguaribe river, in northeastern Brazil

The relationship between fish size and Hg concentrations may help understanding the difference between habitats. Size-Hg concentrations relationships have been reported for several fish species, particularly carnivores, and including *S. rhombeus*. As for the Hg and methyl-Hg contents, size-Hg relationships are distinct between the two sites. In the reservoir habitat *S. rhombeus* showed a significant negative correlation with, whereas in the fluvial habitat Hg concentrations increase with size (Fig. 2). Unfortunately, our sampling design cannot explain the size difference observed between the two sites.

Dórea et al. (2006) found a similar pattern studying the Hg content in *S. rhombeus* from different seasons in the Rio Negro in the northern Amazon. They found increasing Hg content with size in individuals during the low-water period and decreasing concentrations with size in the high-water period. Notwithstanding the relationships were not statistically significant, although another carnivore, *H. malabaricus*, showed a similar trend. The same mechanisms, i.e., decreasing pH, which favor Hg methylation, and encroaching flooded areas, concentration preys and favoring higher predation rates, also seem associated with the distinct patterns of size-Hg relationship between fluvial, decreasing, and reservoir populations increase in Hg concentrations with size. The larger proportion of methyl-Hg in the reservoir *S. rhombeus* supports this hypothesis.

Another species that shows different Hg-size relationships under river and reservoir environmental conditions is the tilapia (*O. niloticus*). Size and Hg concentrations were statistically equal between the two sampled populations, although the wild population originates from the Cocó river (5.1 ± 2.9 ng.g^−1^ w.w.), a highly eutrophic and contaminated fluvial system in the Metropolitan area of Ceará State Capital, Fortaleza, whereas the farmed tilapia originates from the Castanhão Reservoir (6.6 ± 3.0 ng.g^−1^ w.w.), mostly oligotrophic (Santos et al., 2016). Methyl-Hg concentrations too were not significantly different between the two groups (*p* > 0.05). Notwithstanding the Hg-size relationships were different between sites.

**Fig. 3:**
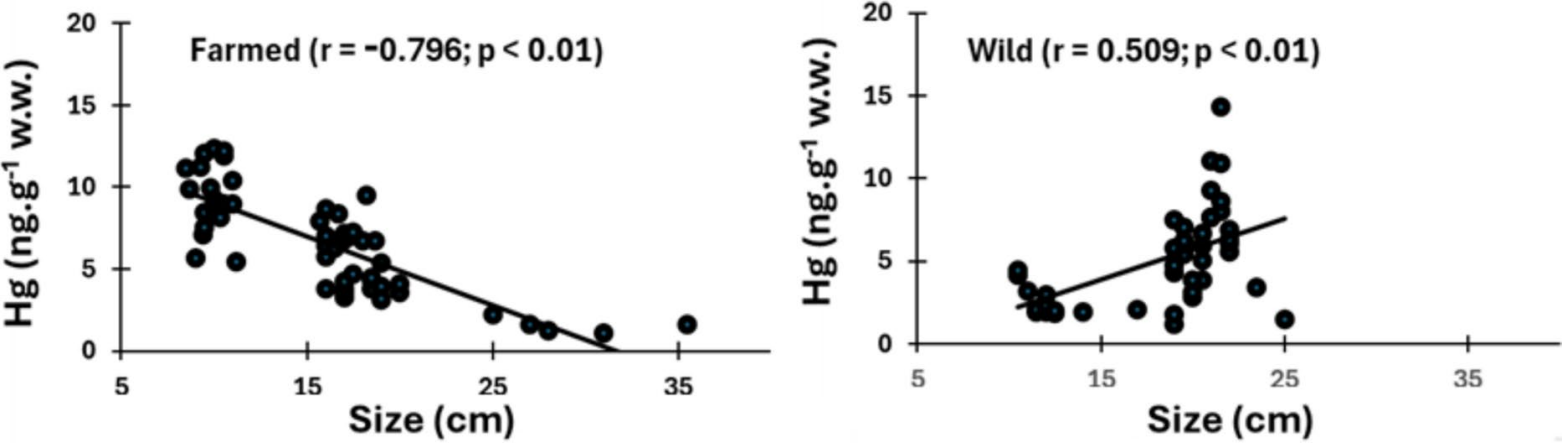
Relationship between size and Hg concentrations in farmed and wild *O. niloticus* sampled in the Castanhão Reservoir and downstream in the Cocó river, in northeastern Brazil

Concentrations of Hg observed in *O. niloticus* from northeastern Brazil are in the same range of most reported contents in wild and farmed Tilapia, which are mostly very low (< 100 ng.g^−1^ w.w.) with few exceptions in highly contaminated sites (Morgado et al., 2005). Ouédraogo & Amyot, 2013; Ouédraogo et al. 2025) reported Tilapia Hg concentrations from 10 sub-Saharan reservoirs to vary from 1 to 76 ng.g^−1^ w.w., while Lacerda et al. (2018) reported 2.7–38.1 ng.g^−1^ w.w., in farmed *O. niloticus* and 17.1–57.3 ng.g^−1^ w.w. in wild ones, also in the Castanhão Reservoir. Notwithstanding the similar concentrations found in wild and farmed Tilapia, however, the relationship between size and Hg concentrations of fish is completely opposite between the two populations (Fig. 3).

Previous comparisons between wild and farmed fish have shown contrasting results. For example, Hg content in farmed tuna remained below the legal limit (1,000 ng.g^−1^ w.w.), whereas in wild tuna concentrations were well over it (1,700 ng.g^−1^ w.w.) (Annibaldi et al., 2019). Individuals of *O. niloticus* also presented higher Hg contents than farmed individuals with similar sizes. However, farmed salmon presented significantly lower Hg concentrations than wild salmon of similar size and the difference is attributed to dilution in rapidly growing farmed fish and to controlled diets (Jardine et al., 2009). In *O. niloticus* from Lake Kariba, Zambia, non-essential metals concentrations, including Hg, were higher in wild fish from areas not affected by the fish farms, except for highly polluted sites. The higher Hg level in wild Tilapia was also associated with the life span of wild and farmed fish. While wild Tilapia can live up to 9 years, farmed ones are typically harvested within 6 months of cage-rearing. Therefore, wild Tilapia can bioaccumulate Hg over a longer life-span (Simukoko et al., 2022). In northeastern Brazil, cage-reared Tilapia, reach market size (0.8 to 1.2 kg and > 20 cm total length in 6 to 7 months) (Nunes & Rocha, 2015) and growth dilution due to short life span seems to explain the decreasing Hg concentrations with size, while longer life span and foraging of wild Tilapia would explain the inverse relationship between Hg concentrations and fish size, as observed in Fig. 2. It is interesting to note that commercialized farmed Tilapia (size > 20 cm) presents the lowest Hg concentrations, whereas the smaller size of wild fish (mostly of size between 15 and 25 cm) results in the highest Hg concentrations. Notwithstanding in both fishes Hg concentrations are extremely low.

### Mercury exposure risk through fish consumption

Exposure estimates showed no significant risk of mercury exposure for fish consumers. In all consumption scenarios and for all consumer groups, estimated Hg ingestion rate (TIHg) was lower or equivalent to the Hg reference dose (RfD = 0.0001 mg.kg^−1^·day^−1^) (Table 2). This result highlights the very low levels observed in fish species from these areas, which result in no risk of exposure regardless of the consumption scenario and/or consumer group. As expected, the calculated maximum safe fish intake was higher than the local per capita fish consumption (FC_NE_), the WHO recommended fish consumption (FC_WHO_) and subsistence consumption rate (FC_subs_), which means there were no scenarios where fish consumption could lead to excessive exposure to Hg. Therefore, we found no need to recommend any limit to the number of monthly fish meals and these amounts were always greater than 30 meals per month (Table 3). It is worth noting that our sampling design did not include collections for all seasons and, thus, this result should be taken with caution.

**Table 2:** Estimated daily ingestion (EDI) (mg kg day ^−1^) for each consumer group (WRA—women of childbearing age; CH—children under 12 years old; AM—adult men over 18 years old; AW—adult women over 46 years old) at each consumption scenario (FC). *EDI > RfD

		FC_NE_ = 0.0245 kg.day^−1^				FC_WHO_ = 0.0285 kg.day^−1^				FC_SUBS_ = 0.142 kg.day^−1^		
Species	WRA^E-06^	CH^E-05^	AM^E-06^	AW^E-06^	WRA^E-06^	CH^E-05^	AM^E-06^	AW^E-06^	WRA^E-05^	CH^E-05^	AM^E-05^	AW^E-05^
*Astronotus ocellatus*	6.29	1.54	5.36	6.12	7.31	1.79	6.23	7.12	3.64	8.91	3.10	3.55
*Cichla kelberi*	3.55	0.87	3.03	3.46	4.13	1.01	3.52	4.02	2.06	5.05	1.76	2.00
*Cichla pinima*	2.84	6.96	2.42	2.77	3.31	0.81	2.82	3.22	1.65	4.03	1.41	1.60
*Cichla sp*	0.12	0.2	10.2	11.6	13.9	3.40	11.8	13.5	6.93	16.9*	5.90	6.74
*Hoplias malabaricus*	4.36	1.07	3.72	4.24	5.07	1.24	4.32	4.94	2.53	6.18	2.15	2.46
*Hoplosternum littorale*	7.50	1.83	6.39	7.30	8.73	2.13	7.44	8.49	4.35	10.6*	3.70	4.25
*Hypostomus pusarum*	1.82	14.4	1.55	1.77	2.11	0.52	1.80	2.06	1.05	2.57	8.96	1.02
*Leporinus friderici*	0.11	2.69	9.38	1.07	1.28	3.13	10.9	12.5	6.38	15.6*	5.43	6.21
*Oreochromis niloticus*	2.50	0.61	2.13	2.43	2.91	0.71	2.48	2.83	1.45	3.54	1.23	1.41
*Plagioscion squamossimus*	0.10	2.49	8.68	9.91	11.9	2.90	10.1	11.5	5.90	14.4	5.03	5.75
*Prochilodus argenteus*	2.62	6.40	2.23	2.55	3.05	0.75	2.60	2.96	1.52	3.71	1.29	1.48
*Prochilodus brevis*	5.53	1.35	4.71	5.38	6.43	1.57	5.48	6.26	3.20	7.83	2.73	3.12
*Prochilodus rubrotaeniatus*	4.86	1.19	4.14	4.73	5.65	1.38	4.82	5.50	2.82	6.89	2.40	2.74
*Serrasalmus rhombeus*	9.86	2.41	8.41	9.60	11.5	2.81	9.78	11.2	5.72	14.0*	4.87	5.56

**Table 3:** Maximum safe fish intake (FC_máx_) (kg day^−1^) and the recommended safe monthly fish meals (FC_meals_) (meals month^−1^) for each consumer group (WRA—women of childbearing age; CH—children under 12 years old; AM—adult men over 18 years old; AW—adult women over 46 years old)

Species	Location	FC_máx_				FC_meals_			
		WRA	CH	AM	AW	WRA	CH	AM	AW
*Astronotus ocellatus*	Açude Banabuiú	0.39	0.16	0.46	0.40	79	65	93	81
*Cichla kelberi*	Açude Castanhão	0.69	0.29	0.80	0.71	140	114	164	144
*Cichla pinima*	Açude Castanhão	0.86	0.35	1.01	0.88	175	143	205	180
*Cichla sp*	Rio Jaguaribe	0.20	0.08	0.24	0.21	42	34	49	43
*Hoplias malabaricus*	Rio Jaguaribe; Açude Castanhão	0.56	0.23	0.66	0.58	114	93	134	117
*Hoplosternum littorale*	Rio Cocó	0.33	0.13	0.38	0.34	66	54	78	68
*Hypostomus pusarum*	Rio Cocó	1.35	0.55	1.58	1.39	274	224	321	281
*Leporinus friderici*	Rio Jaguaribe	0.22	0.09	0.26	0.23	45	37	53	46
*Oreochromis niloticus*	Rio Jaguaribe; Itaiçaba; Açude Banabuiú; Aquicultura; Açude Castanhão	0.98	0.40	1.15	1.00	199	163	233	204
*Plagioscion squamosissimus*	Açude Castanhão	0.24	0.10	0.28	0.25	49	40	57	50
*Prochilodus argenteus*	Rio Jaguaribe	0.94	0.38	1.10	0.96	190	155	223	195
*Prochilodus rubrotaeniatus*	Rio Cocó	0.50	0.21	0.59	0.52	102	84	120	105
*Prochilodus brevis*	Açude Banabuiú	0.44	0.18	0.52	0.46	90	74	106	93
*Serrasalmus rhombeus*	Açude Castanhão	0.25	0.10	0.29	0.26	50	41	59	52

The fish safe level (FSL) estimated for each consumption rate scenario varied according to the consumer group (Table 4) and was lower for children compared to other groups. The FSL parameter is the Hg concentration limit, in the fish, that would warrant further investigation if exceeded in order to avoid the risk of excessive exposure to Hg from consuming seafood. Our results were comparable to the fish tissue residue criterion (TRC) established for freshwater and estuarine fishes by USEPA (2001) and reported by Bezerra et al. (2023). We found only a few species exceeding the lowest calculated FSL value, which was estimated for children consuming fish at a high subsistence rate. Still, these were only marginally higher and, thus, they present no risk to these consumers. Fish safe levels (FSLs) are very sensitive not only to the chosen fish consumption rate, but also to the consumer body weight, which is why it varies across the studied scenarios. The FSL value should be used as an a priori screening when monitoring Hg contamination in seafood and should not substitute the established 1 mg kg^−1^ (or 0.5 mg kg^−1^ for non-predator fish) concentration limit established by many governmental institutions.

**Table 4:** Safe fish screening Hg level (ng g ^−1^) for each scenario and consumer group. Hg concentrations at this level in consumed fish likely pose no risk of Hg exposure

Consumer	FC_NE_ = 0.0245	FC_WHO_ = 0.0285	FC_SUBS_ = 0.142
Women of childbearing age (18 to 45 years old)	237.6	204.2	41.0
Children (1 to 12 years old)	97.1	83.5	16.8
Adult males (over 18 years old)	278.8	239.6	48.1
Adult women (over 46 years old)	244.1	209.8	42.1

These results were corroborated by the calculated THQ index that represents the risk of chronic exposure to non-carcinogenic contaminants, where a THQ < 1 means no potential risk of adverse effects and for THQ > 1 there is a potential risk of adverse effects in consumers. We found THQ values below 1 for all species, in all consumption scenarios, and for all consumer groups (Tab. 5). Even when considering the high consumption (in a subsistence basis), by children, of carnivorous/omnivorous fish species, including *Cichla* sp.*, Plagioscion squamossimus, Serrasalmus rhombeus, and Leporinus friderici*, our estimates showed a negligible risk of excessive exposure to Hg (Tables 3 and 5). These results contrast those by Bezerra et al. (2023) and Matias-Nogueira et al. (2025), which showed that exposure risk from the consumption of marine fish in Ceara is strongly dependent on the species consumed, especially in upper-level/large-bodied species, including sharks and tunas.

**Table 5:** Estimated species Target Hazard Quotient (THQ) for each consumer group (WRA—women of childbearing age; CH—children under 12 years old; AM—adult men over 18 years old; AW—adult women over 46 years old) at each consumption scenario (FC)

	FC_NE_ = 0.0245 kg.day^−1^				FC_WHO_ = 0.0285 kg.day^−1^				FC_SUBS_ = 0.142 kg.day^−1^			
Species	WRA	CH	AM	AW	WRA	CH	AM	AW	WRA	CH	AM	AW
*Astronotus ocellatus*	0.06	0.15	0.05	0.06	0.07	0.18	0.06	0.07	0.36	0.89	0.31	0.35
*Cichla kelberi*	0.04	0.09	0.03	0.03	0.04	0.10	0.04	0.04	0.21	0.50	0.18	0.20
*Cichla pinima*	0.03	0.07	0.02	0.03	0.03	0.08	0.03	0.03	0.16	0.40	0.14	0.16
*Cichla *sp.	0.12	0.29	0.10	0.12	0.14	0.34	0.12	0.14	0.69	1.69	0.59	0.67
*Hoplias malabaricus*	0.04	0.11	0.04	0.04	0.05	0.12	0.04	0.05	0.25	0.62	0.22	0.25
*Hoplosternum littorale*	0.08	0.18	0.06	0.07	0.09	0.21	0.07	0.08	0.43	1.06	0.37	0.42
*Hypostomus pusarum*	0.02	0.04	0.02	0.02	0.02	0.05	0.02	0.02	0.11	0.26	0.09	0.10
*Leporinus friderici*	0.11	0.27	0.09	0.11	0.13	0.31	0.11	0.12	0.64	1.56	0.54	0.62
*Oreochromis niloticus*	0.02	0.06	0.02	0.02	0.03	0.07	0.02	0.03	0.14	0.35	0.12	0.14
*Plagioscion squamossimus*	0.10	0.25	0.09	0.10	0.12	0.29	0.10	0.12	0.59	1.44	0.50	0.57
*Prochilodus argenteus*	0.03	0.06	0.02	0.03	0.03	0.07	0.03	0.03	0.15	0.37	0.13	0.15
*Prochilodus brevis*	0.06	0.14	0.05	0.05	0.06	0.16	0.05	0.06	0.32	0.78	0.27	0.31
*Prochilodus rubrotaeniatus*	0.05	0.12	0.04	0.05	0.06	0.14	0.05	0.06	0.28	0.69	0.24	0.27
*Serrasalmus rhombeus*	0.10	0.24	0.08	0.10	0.11	0.28	0.10	0.11	0.57	1.40	0.49	0.56

## Conclusion

This is the first survey of Hg contamination and exposure risk to fish consumers in this region. Methyl-Hg and Hg concentrations were quantified in 14 fish species, representing the most typical products of inland fisheries and aquaculture in northeastern Brazil. Our results showed very low contamination levels and Hg concentrations in all individuals were well below the limits established by the local environmental and sanitary legislation. Concentrations are also much lower than reported values from other regions worldwide, and in the tropical America in particular. There were no significant differences in Hg accumulation among the sampled species, but for some carnivorous fish, which showed significantly higher Hg concentrations. Some of these carnivores also showed higher methyl-Hg concentrations. Mercury chemical species concentrations were relatively higher in individuals from fluvial sites compared to reservoirs. We observed a contrasting pattern in fish size-Hg relationships between fluvial and reservoir habitats. Total Hg concentrations were observed to increase with fish body size in fluvial, but not in reservoir habitats. On the other hand, in reservoirs and farmed fish total Hg concentrations showed a negative correlation with size, with lower Hg concentrations in larger individuals, suggesting growth dilution effect (in farmed species). These farmed fish, which display the lowest Hg levels, fall within the size range of commercialized individuals. As a result, the estimates of exposure and risk to consumers suggest as recommendations that even with daily intake of any species analyzed, their consumption is safe, from the point of view of exposure to Hg, particularly the farmed Tilapia, the major aquaculture product in the region.

## Data Availability

All results are available upon request from the corresponding author.
